# *Deparia × nanakuraensis* K.Hori (Athyriaceae), a new hybrid pteridophyte from Japan

**DOI:** 10.3897/phytokeys.165.57837

**Published:** 2020-10-28

**Authors:** Kiyotaka Hori

**Affiliations:** 1 The Kochi prefectural Makino Botanical Garden, Kochi, Japan The Kochi prefectural Makino Botanical Garden Kochi Japan

**Keywords:** Athyriaceae, *
Deparia
*, new hybrid, Japan

## Abstract

I describe Deparia × nanakuraensis**hyb. nov.** and discuss differences in morphological characteristics between parental species *D. pterorachis* and *D. viridifrons* with chromosome counting, plastid, and nuclear DNA markers. The new hybrid is endemic to the eastern and northern parts of Japan. Based on the criteria of the International Union for Conservation of Nature and Natural Resources, this new species is here considered Data Deficient. The ploidy level is diploid sterile.

## Introduction

The genus *Deparia* Hook. & Grev. is one of the largest groups in the Athyriaceae family. It contains 60–90 species mostly in East Asia, with some species distributed in Africa, western Indian Ocean, northeastern North America, the Hawaiian Islands, Australia, New Zealand, and South Pacific Islands (Kato 1984; Rothfels et al. 2012; He et al. 2013; Kuo et al. 2016, 2018; PPG I 2016; Moran et al. 2019).

The genus is characterized by hair-like scales and disconnected grooves between rachises and costae (Kato 1973, 1977, 1984; Rothfels et al. 2012; Sundue and Rothfels 2014; Kuo et al. 2018). These two features have not been observed in the genera *Anisocampium* C.Presl, *Athyrium* Roth., *Diplazium* Sw., *Ephemeropteris* R.C.Moran & Sundue, and *Pseudathyrium* Newman but in some species in the Athyriaceae family (Kato 1973; Rothfels et al. 2012; Moran et al. 2019). In addition, narrowly U-shaped rachis grooves are also a unique character of the genus *Deparia* (Kuo et al. 2018). The basic chromosome number of *Deparia* is 40, contrary to *Diplazium* of 41 (Sano et al. 2000; Rothfels et al. 2012).

In Japan, several hybrids of the genus *Deparia* have been described: D. × birii Fraser-Jenk. (Fraser-Jenkins 2008), D. × kiyozumiana (Sa.Kurata) Y.Shimura (Shimura 1980), pentaploid sterile *D. lancea* (Thunb.) Fraser-Jenk. (Nakato and Mitui 1979), D. × lobatocrenata (Tagawa) M.Kato (Kato 1984; Ebihara 2017), D. × musashiensis (H.Ohba) Seriz. (Serizawa 1981), pentaploid sterile *D. petersenii* (Kunze) M.Kato (Shinohara et al. 2003), D. × togakushiensis Otsuka & Fujiw. (Otsuka and Fujiwara 1999), and D. × tomitaroana (Masam.) R.Sano (Sano et al. 2000). Furthermore, Ebihara (2017) mentioned several combinations of hybrids that are not still described.

The *Deparia okuboana* complex (Athyriaceae) is recently defined by Ebihara (2017) as consisting of *D. okuboana* (Makino) M.Kato (apogamous triploid; Hirabayashi 1970), *D. coreana* (Christ) M.Kato (sexual tetraploid, Nakato and Ebihara 2018), *D. henryi* (Baker) M.Kato (apogamous triploid, Nakato and Ebihara 2018), *D. viridifrons* (Makino) M.Kato (sexual diploid; Hirabayashi 1970), *D. unifurcata* (Baker) M.Kato (apogamous triploid; Hirabayashi 1970), *D. pterorachis* (Christ) M.Kato (sexual diploid; Hirabayashi 1970). There is continuous morphological variation between *D. coreana*, *D. henryi*, *D. okuboana*, and *D. unifurcata* (Ebihara 2017). Kuo et al. (2018) identified that these members belong to sect. Dryoathyrium. Hori (2018) reported there were reticulate relationships in the *D. okuboana* complex with sect. Lunathyrium (Kuo et al. 2018) based on plastid and nuclear DNA marker. In addition, Ebihara (2017) mentioned undescribed diploid sterile hybrid between *D. pterorachis* and *D. viridifrons* based on morphology and ploidy level. This study described this new hybrid of *D. pterorachis* and *D. viridifrons*, Deparia × nanakuraensis K.Hori, based on morphological characteristics, chromosome number, plastid, and nuclear DNA marker.

## Materials and methods

### Plant materials, Chromosome count, and DNA extraction

In this study, *Deparia viridifrons*, D. × nanakuraensis, and *D. pterorachis* were investigated in molecular DNA analysis. Other members of the *D. okuboana* complex (*D. coreana*, *D. henryi*, *D. okuboana*, *D. unifurcata*) and Japanese members of the sect. Lunathyrium (D. pycnosora var. albosquamata M.Kato, D. pycnosora (Christ) M.Kato var. pycnosora, D. pycnosora var. mucilagina M.Kato) were also used as materials. Voucher information for all samples is listed in Appendix I. All voucher specimens have been deposited in the Makino Herbarium of Tokyo Metropolitan University (MAK), and/or the Kochi Prefectural Makino Botanical Garden (MBK). The DNA sequences of *Athyrium melanolepis* Christ, *A. crenulatoserrulatum* Makino, *A. opacum* Copel., *Diplazium chinense* (Baker) C.Chr., *Di. esculentum* (Retz.) Sw., *Di. wichurae* (Mett.) Diels were used as outgroups, quoted from the Genbank database.

Additionally, specimens from the Collection Database and Materials of TNS (http://db.kahaku.go.jp/webmuseum/), PE (http://pe.ibcas.ac.cn/en/), TAIF (http://taif.tfri.gov.tw/search.php), and from the JSTOR Global Plants (https://plants.jstor.org/) as well as from the Global Biodiversity Information Facility (GBIF: https://www.gbif.org) database were checked.

For the conservation assessment, the area of occupancy (AOO) and extent of occurrence (EOO) were estimated using GeoCAT (Bachman et al. 2011), default settings for grid size were applied. In addition, mitotic chromosomes from D. × nanakuraensis were counted.

To observe mitotic chromosomes, root tips were collected in the field, and pre-treated with 0.004 M 8-hydroxyquinoline for 6 h at approximately 17–20 °C. After fixation in ethanol and acetic acid (3:1) for 15–30 min, the root tips were hydrolyzed in 1 N HCl at 60 °C for 1–3 min and then squashed in 2% aceto-orcein solution. The chromosomes were observed under a microscope (Leica DM2500) and then photographed by using a digital camera (Leica MC170 HD).

For the molecular analyses, total DNA was extracted from silica-dried leaves using cetyltrimethylammonium bromide solution, according to Doyle and Doyle (1990).

### Plastid and nuclear DNA sequencing

*trnL-F* was used as the maternally-inherited (Gastony and Yatskievych 1992; Kuo et al. 2018) plastid DNA marker (F: 5'-ATTTGAACTGGTGACACGAG-3' and FernL 1 Ir1: 5'-GGYAATCCTGAGCAAATC-3'; Taberlet et al. 1991; Li et al. 2009). *AK1* (AK4F: 5'-GATGAAGCCATCAAGAAACCA-3' and AKR2: 5'-ATGGATCCAGCGACCAGTAA-3'; Hori and Murakami 2019) was used as a biparentally-inherited nuclear marker for polymerase chain reaction-single-strand conformation polymorphism (PCR–SSCP) analysis, which was used to determine allelic variation in each individual (Hori and Murakami 2019).

PCR amplification was performed using PrimeSTAR Max DNA Polymerase (Takara, Kyoto, Japan). PCR entailed an initial denaturation step at 95 °C for 10 min, followed by 35 cycles of denaturation, annealing, and elongation steps at 98 °C for 10 s, 55 °C for 5 s, and 72 °C for 5 s, respectively, using a Model 9700 thermal cycler (Applied Biosystems, Foster City, CA, USA).

Gel electrophoresis of *AK1*PCR products was performed using gels of 50% MDE gel solution (Lonza) containing 2% glycerol at 15 °C for 16 h at 300 V, followed by silver staining. For sequencing of the bands separated on the gels, the polyacrylamide gel was dried after silver staining by sandwiching the gel between Kent paper and a cellophane sheet on an acrylic backplate at 55 °C for 4 h. To extract the DNA, a piece of the DNA band was peeled from the dried gel using a cutter knife and incubated in 50 μL of Tris-EDTA buffer (10-mM Tris-HCl and 1-mM EDTA, pH 8.0) at 4 °C overnight. The supernatant solution was used as a template for further PCR amplification with the same primer set employed for initial PCR amplification.

PCR products were purified using Illustra ExoStar 1-Step (GE Healthcare, Wisconsin, USA) and used as templates for direct sequencing. Reaction mixtures for sequencing were prepared using the BigDye Terminator v.3.1 Cycle Sequencing Kit (Applied Biosystems). The reaction mixtures were analyzed using an ABI 3130 Genetic Analyzer (Applied Biosystems).

### Molecular analysis

The accession numbers of DNA sequences in the datasets were shown in Appendix I. The sequences were aligned using MUSCLE (Edgar 2004) and assessed with Bayesian inference (BI) analysis using MrBayes 3.2.6 (Ronquist et al. 2012), maximum parsimony (MP), and maximum likelihood (ML) analysis using the MEGA X software (Kumar et al. 2018). Indels were treated as missing characters in all analyses. In the BI analysis, the best-fit model (*trnL-F*: HKY+I model; *AK1*: HKY model) of sequence evolution for each DNA region was selected using jModelTest 2.1.10 (Darriba et al. 2012). Four Markov chain Monte Carlo chains were run simultaneously and sampled every 100 generations for 1 million generations in total. Tracer 1.7.1 (Rambaut et al. 2018) was used to examine the posterior distribution of all parameters and their associated statistics, including estimated sample sizes. The first 2,500 sample trees from each run were discarded as burn-in periods. The MP tree was obtained using the Tree-Bisection-Regrafting (TBR) algorithm (Nei and Kumar 2000) at search level 3, at which the initial trees were obtained by the random addition of sequences (100 replicates). The confidence level of the monophyletic groups was estimated with 1,000 MP bootstrap pseudo-replicates. In ML analysis, the best-fitting model of sequence evolution for each marker was selected using MEGA; Tamura 3-parameter + I model was used for *trnL-F* and HKY model for *AK1*. Initial trees for the heuristic search were obtained automatically by applying Neighbor-Join and BioNJ algorithms to a matrix of pairwise distances estimated using the Maximum Composite Likelihood approach and then selecting the topology with superior log likelihood value. The bootstrap method with 1,000 replications was employed to estimate the confidence levels of monophyletic groups in MP and ML analysis.

## Results

### Chromosome count

**Figure 1.**
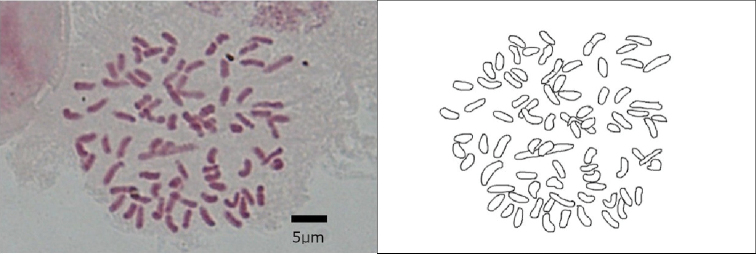
Photograph and sketch of mitotic metaphase chromosomes (2*n* = 80) of D. × nanakuraensis (*Hori 3391*).

Mitotic metaphase chromosome number observed in an individual of D. × nanakuraensis (*Hori 3391*) was 2*n* = 80 (Figure 1). This individual had shrunken sporangium with no spores. The basic chromosome numbers of the genus *Deparia* is *x*= 40 (Sano et al. 2000; Rothfels et al. 2012), and suitably, this sample was found to be a sterile diploid.

### Plastid and nuclear DNA phylogenetic trees

We sequenced 653–746 bp of the *trnL-F* intergenic spacer from different specimens. The aligned *trnL-F* matrix was 765 bp, of which 121 characters (15%) were parsimony-informative. For the *AK1* intron, we sequenced 338–590 bp of the intron for each specimen, yielding a 604 bp aligned matrix, of which 74 characters (12%) were parsimony-informative.

**Figure 2.**
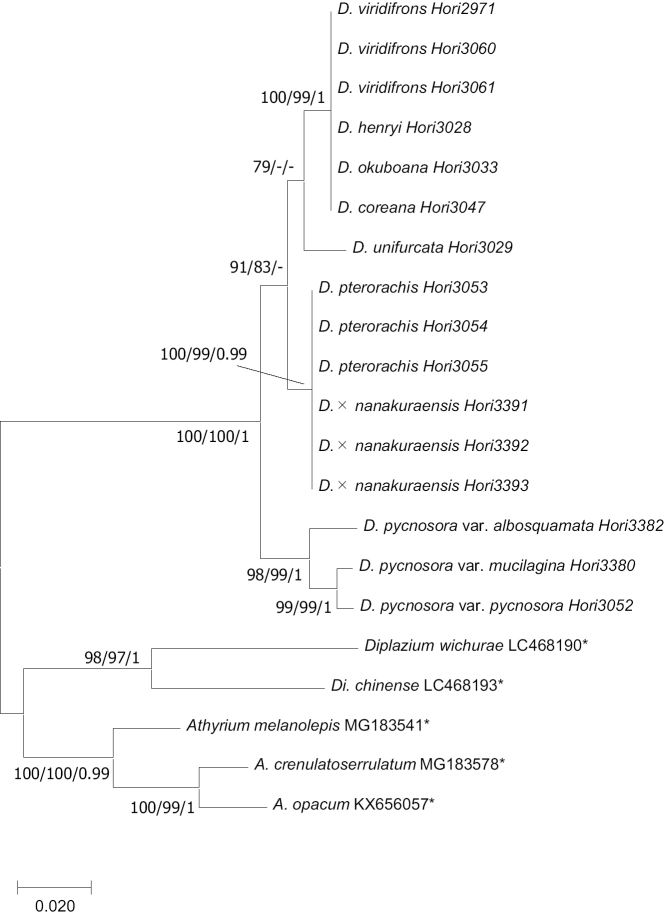
The ML tree based on the sequence variation of the gene *trnL-F* (ln *L* = -2309.05) with PP (>0.90) and BP (>70) of ML/MP/BI analyses on each branch. The sequences with asterisks were quoted from Genbank.

**Figure 3.**
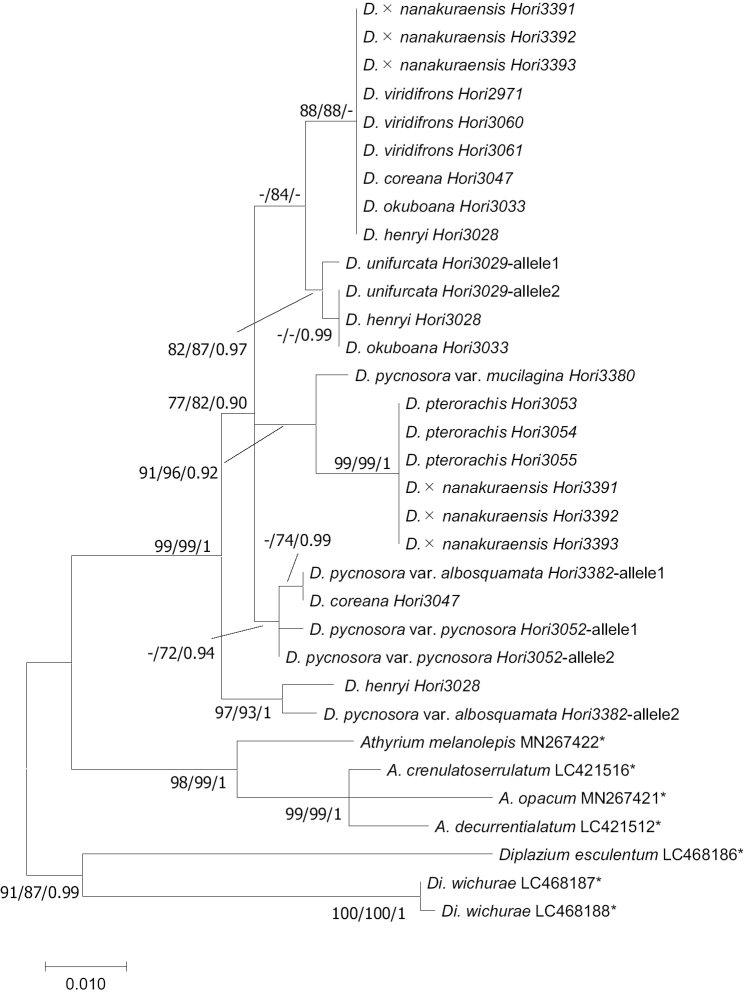
The ML tree based on the sequence variation of the gene *AK1* (ln *L* = -1616.59) with PP (>0.90) and BP (>70) of ML/MP/BI analyses on each branch. The sequences with asterisks were quoted from Genbank.

The ML trees according to the sequences of *trnL-F* (ln *L* = -2309.05) and *AK1* (ln *L* = -1616.59) with bootstrap percentages (BPs), Bayesian posterior probabilities (PP) were shown in Figures 2, 3, respectively. In the *trnL-F* phylogeny, the haplotype of *D. pterorachis* and *D. viridifrons* composed different clades with *D. coreana*, *D. henryi*, and *D. okuboana* which were supported by BP (>70) and PP (>0.90) values. In the *AK1* phylogeny, the two clades containing *D. pterorachis* and *D. viridifrons* were supported by BP, but *D. viridifrons* was not supported by PP value. Deparia × nanakuraensis had the same haplotype of *D. pterorachis* and *D. viridifrons* in both *trnL-F* and *AK1* phylogenies. Other members of the *D. okuboana* complex (*D. coreana*, *D. henryi*, *D. okuboana*, *D. unifurcata*) shared the same alleles with *D. viridifrons* partly (Hori 2018), but the combination of alleles was different from D. × nanakuraensis. Japanese members of the sect. Lunathyrium (D. pycnosora var. albosquamata, D. pycnosora var. pycnosora, D. pycnosora var. mucilagina) did not share any alleles with D. × nanakuraensis. Therefore, D. × nanakuraensis can be of origin hybrid from *D. pterorachis* and *D. viridifrons*.

### Taxonomic treatment

#### Deparia × nanakuraensis

Taxon classificationPlantaePolypodialesAthyriaceae

K.Hori hyb. nov.

CEDDEB9E-C4B4-5EF9-AF13-6A8F36F3735F

urn:lsid:ipni.org:names:77212571-1

Figure 4

##### Type.

Japan. Honshu: Akita prefecture, Noshiro city, Futatsui town, Nanakura-shrine, 40°12'9.48"N, 140°15'29.82"E, alt. 23 m, deciduous forest containing *Acer miyabei* Maxim., *Aesculus turbinata* Blume, *Cercidiphyllum japonicum* Siebold & Zucc., *Cryptomeria japonica* (Thunb. ex L.f.) D.Don, *Dryopteris monticola* (Makino) C.Chr., and *Pachysandra terminalis* Siebold & Zucc., on soil, 7 Jul 2020, *K. Hori 3391* (holotype: MAK467056; isotype: MBK).

**Figure 4.**
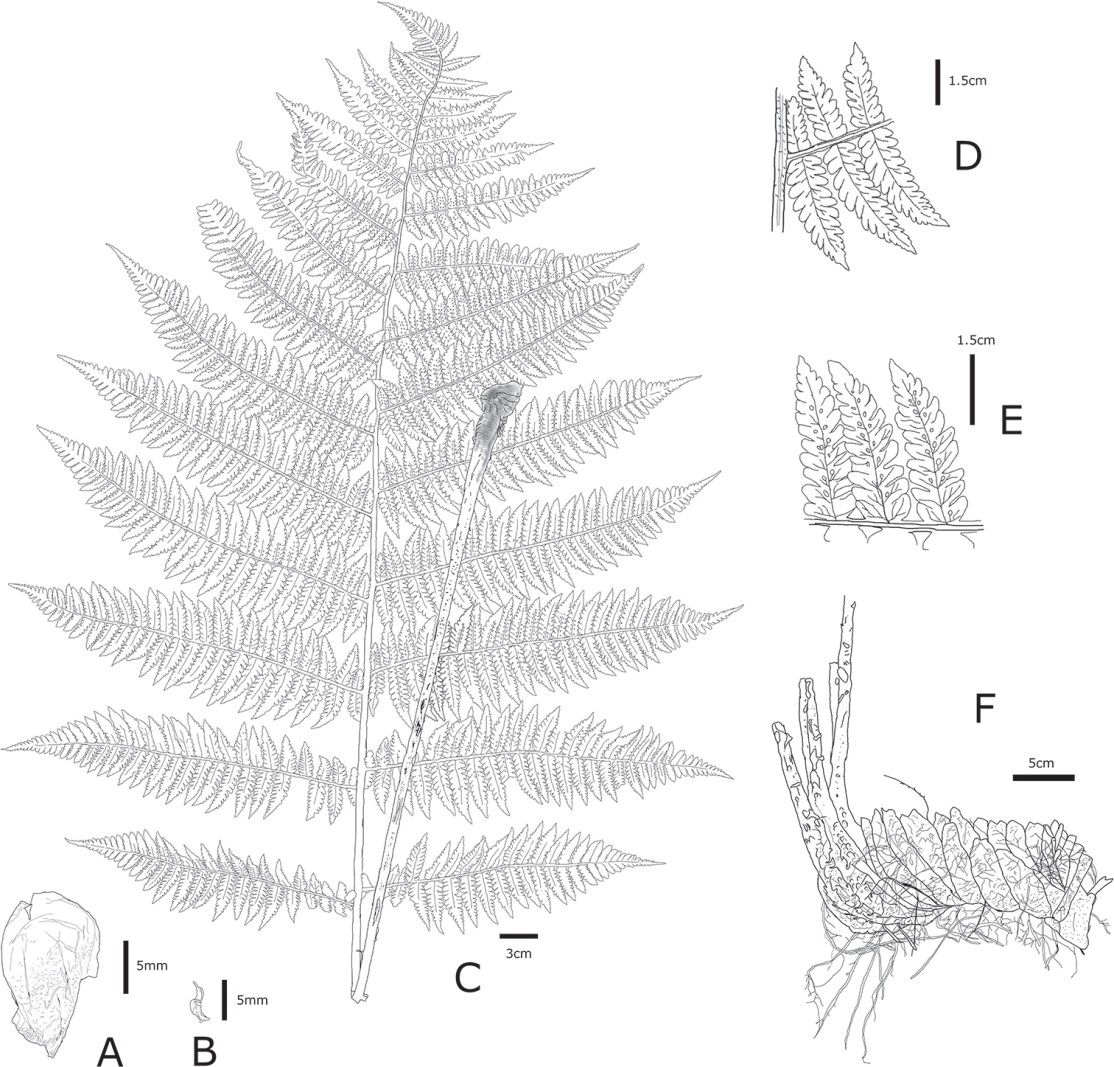
D. × nanakuraensis K.Hori **A** lower stipe scale **B** upper stipe scale **C** abaxial surface of frond and stipe **D** detail of adaxial pinnule **E** detail of abaxial pinnule, and **F** rhizome and base of stipes. **A–F** from the holotype (MAK467056) (illustration by K. Hori).

##### Description.

*Terrestrial, summer green fern. Rhizomes* creeping, occasionally branched, with buds, stramineous, 15–25 × 4–7 cm, closely set with roots and persistent, densely clothed by old stipe bases, glabrous; *fronds* 4–6 per rhizome; *stipes* whitish green, 30–40 × 0.8–1.5 cm, sparsely clothed with stramineous scales at the base (1–1.5 × 0.5–1 cm), ovate; *blades* yellowish green adaxially, 3-pinnate-pinnatifid at the base, in the middle to upper section, 2-pinnate at the apex, 40–70 × 30–40 cm, deltoid; *rachises* whitish green, glabrous, sparsely clothed with stramineous scales (2–5 × 1–2 mm) and black hairs adaxially; *pinnae* 10–15 pairs, ascending, lanceolate, shrunken at base, alternate, petiolated (2–5 mm), sessile near the apex, lowest pinnae slightly reduced, second lowest pair usually the largest, 25–30 × 4–8 cm; *pinnules*, alternate on the basal and middle sections of the blade, 20–30 pairs on the basal and middle sections of the blade, 15–20 pairs on the apex of the blade, reduced distally, lanceolate, deeply serrated, vein-free, close to or reaching to the margin, 10–15 pairs in the middle lobe; *sori* brown, tending to appear on the abaxial surface of the middle or upper part of blades, oblong- to J-shaped, 1.5–3 mm long, on the apex or middle of veinlets, 5–10 per ultimate segment, persistent; *indusium* entire to serrated on margin, *sporangium* shrunken, spores absent.

##### Etymology.

The name derives from Nanakura-shrine, Futasui town, Noshiro City, Akita prefecture, northeast Japan, where Deparia × nanakuraensis was first found.

##### Specimens examined.

**Japan. Honshu**: Akita pref., Noshiro city, Futatsui town, Nanakura-shrine, 40°12'9.48"N, 140°15'29.82"E, alt. 23 m, 7 Jul 2020, *K. Hori 3392, loc. cit., K. Hori 3393, loc. cit., K. Hori 3394, loc. cit.*, 10 Jul 2012, *Y. Horii* 35548 (TNS 01167830), *loc. cit., Y. Horii* 35549 (TNS 01167829); Aomori pref., Hachinohe city, Same town, Kamikoswa, alt. 100 m, 23 Aug 1975, coll. *M. Neichi* (TNS 1170337, image!); *loc. cit.*, Kitsunetai, alt. 30m, 9 Jul 2005, coll. *M. Neichi* (TNS 01183638, image!); Iwate pref., Iwaizumi town, Atsuka, Matsugasawa, alt. 350 m, 18 Jul 1981, coll. *M. Neichi* (TNS 01161869, image!); *loc. cit.*, Ichinoseki city, Higashiyama cho, Nagasaka, Nagahira, alt. 180 m, 22 Aug 1987, coll. *M. Suzuki* (TNS 932028, image!); *loc. cit.*, Maikawa, Ohira, alt. 120 m, 22 Sep 1986, coll. *M. Suzuki* (TNS 9320284image!); Miyagi pref., Ishinomaki city, Mano, Uchihara, alt. 70 m, 25 May 1990, coll. *K. Shogo* (TNS01184195, image!); *loc. cit.*, Sendai city, Akiu town, Baba, alt. 200 m, 15 Oct 1983, coll. *K. Shogo* (TNS01184194, image!); Yamagata pref., Kamiyama city, Takano, alt. 250 m, 5 Jun 1983, coll. *N. Sakawa* (TNS01161877, image!); Fukushima pref., Minamiaizu county, Shimosato town, Yunokami, alt. 500 m, 8 Sep 1972, coll. *T. Waku* (TNS01161873, image!); Saitama pref., Hannnou city, Kasasugitouge, alt. 500 m, 21 Aug 1984, coll. *T. Iwata* (TNS01140142, image!); *loc. cit.*, 14 Sep 1980, coll. *Y. Kobayashi* (MBK0233005); *loc. cit.*, 14 June 1981, coll. *Y. Kobayashi* (MBK0232983).

##### Distribution and ecology.

**Figure 5.**
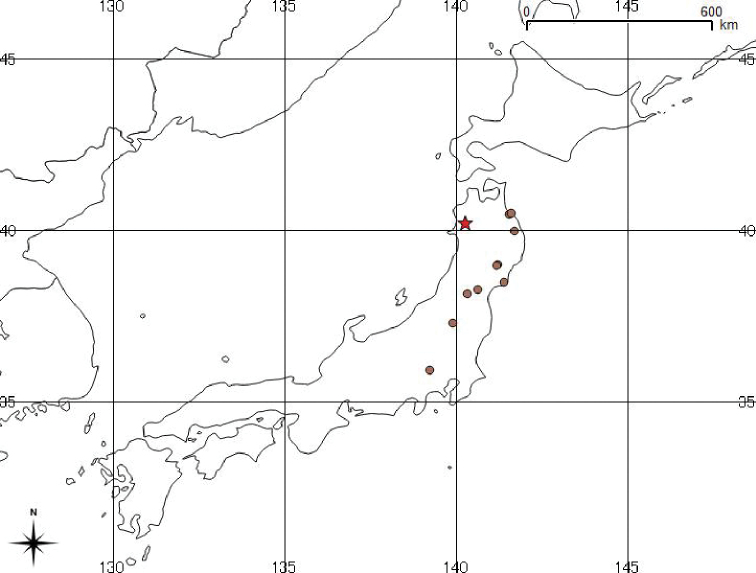
Map showing the known distribution of D. × nanakuraensis in Japan. Red star indicates type locality, other circles indicate examined specimens.

**Figure 6.**
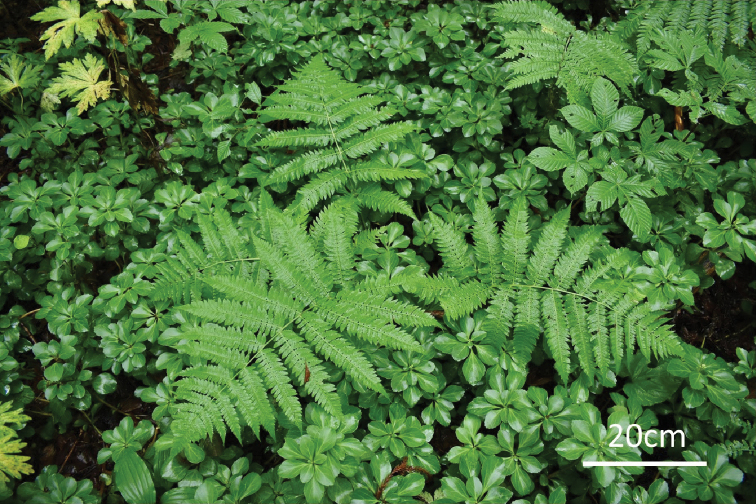
Wild plant of D. × nanakuraensis in type locality.

**Figure 7.**
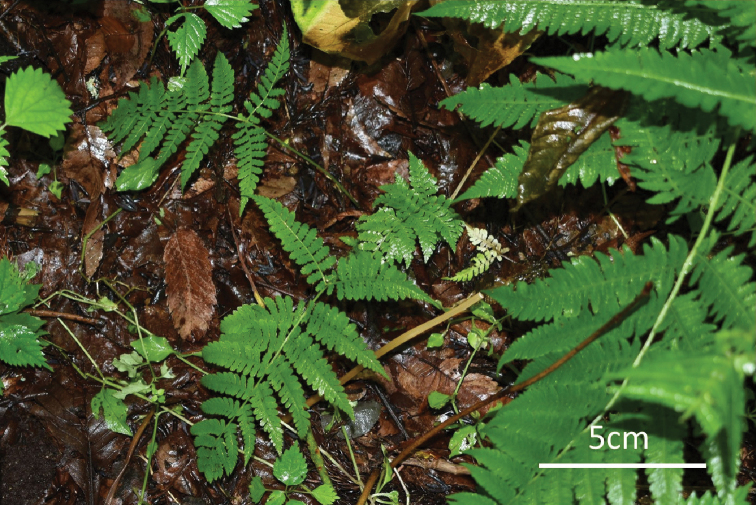
Juvenile of D. × nanakuraensis.

**Figure 8.**
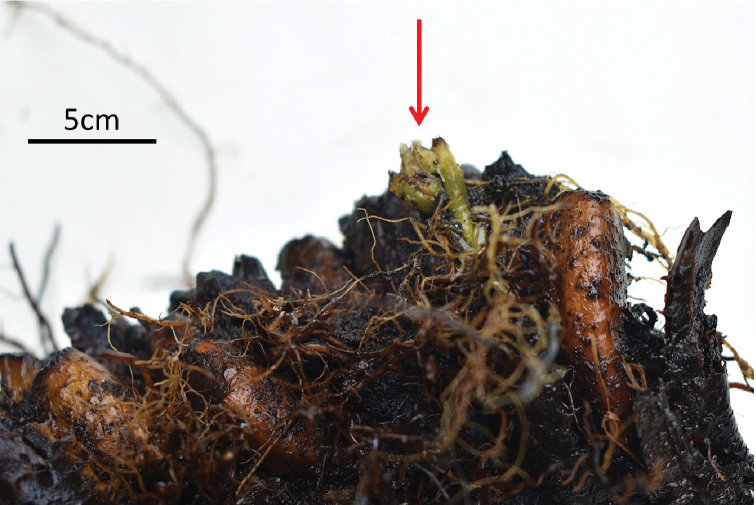
Indefinite growth through bud (red arrow) on rhizome of D. × nanakuraensis.

Deparia × nanakuraensis is known from the eastern and northern part of Honshu in Japan (Figure 5). It was observed to grow on soil under deciduous forest (Figure 6) or planted coniferous forest containing *Cryptomeria japonica*. This hybrid is endemic to Japan. In the type locality, this hybrid comprised a population of over 30 individuals with juveniles (Figure 7) although parents of *D. viridifrons* and *D. pterorachis* were both absent, and sporangium had no spores. However, it is expected that Deparia × nanakuraensis can reproduce young individuals from buds on its rhizome (Figure 8).

##### Conservation status.

IUCN Red List Category. Based on estimates from GeoCAT, the EOO of D. × nanakuraensis was 46,321 km^2^. The known AOO of D. × nanakuraensis was 44 km^2^. The localities correspond to less than 20 points, but I could not check the population size on each locality. Therefore, available information is inadequate to support the assessment of its extinction risk. According to the IUCN (2012) criteria, the category of Data Deficient (DD) is appropriate.

## Discussion

**Figure 9.**
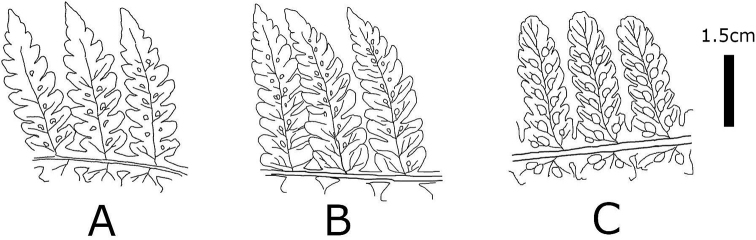
Abaxial surface of pinnule and sori of **A***D. viridifrons***B**D. × nanakuraensis, and **C***D. pterorachis* (illustration by K. Hori).

Deparia × nanakuraensis presents almost intermediate morphologies between *D. viridifrons* and *D. pterorachis* species. *Deparia viridifrons* is characterized by having deltoid-ovate or ovate-lanceolate fronds, reniform to U-shaped sori, pinnules with costal wing, rounded serration of pinnules, and acute apex of pinnules. In contrast, *D. pterorachis* has oblong fronds, oblong to J-shaped sori, pinnules truncated to costa; truncate serration of pinnules, and obtuse apex of pinnules (He et al. 2013; Ebihara 2017). Deparia × nanakuraensis has deltoid fronds, oblong to J-shaped sori, pinnules with narrow costal wing, rather rounded serration of pinnules, and a rather acute apex of pinnules (Figure 9, Table 1).

**Table 1.** Morphological comparison among D. × nanakuraensis and related species.

Characteristics	Shape of frond	Shape of sori	Margin of indusium	Base of pinnule	Serration of pinnules	Apex of pinnules
*D. viridifrons*	deltoid-ovate or ovate-lanceolate	reniform to U-shaped	serrated	with costal wing	rounded	acute
D. × nanakuraensis	deltoid	oblong to J-shaped	entire to serrated	with narrow costal wing	rather rounded	rather acute
*D. pterorachis*	oblong	oblong to J-shaped	entire	truncated to costa	truncate	obtuse

Kuo et al. (2018) classified *D. viridifrons* and *D. pterorachis* as the members of sect. Dryoathyrium because lateral pinnules are not auricled, and these are closely related in plastid DNA phylogeny (Kuo et al. 2018). Therefore, Deparia × nanakuraensis is infra section hybrid in the sect. Dryoathyrium.

The ploidy level of this hybrid is the same as its parents because *D. viridifrons* and *D. pterorachis* are both sexual diploid (Kurita 1963; Mitui 1966, 1968, 1970; Hirabayashi 1970). In addition, this can be the first report of a diploid sterile hybrid of the genus *Deparia* from Japan although several hybrids have been described (Ebihara 2017).

In conclusion, this study described Deparia × nanakuraensis based on morphology, cytology, and molecular DNA analysis. The morphological characteristics were intermediate between its parents *D. viridifrons* and *D. pterorachis*. This hybrid can produce young individuals from buds on its rhizome. Based on the criteria of the International Union for Conservation of Nature and Natural Resources, this new species is here considered Data Deficient. This hybrid can be the first report of diploid sterile hybrid of the genus *Deparia* from Japan. In future studies, it is expected that more hybrids of the genus *Deparia* will be discovered and described from Japan.

## Supplementary Material

XML Treatment for Deparia × nanakuraensis

## Appendix I

**Voucher specimens for DNA analysis in this study.** Data are in the order: Species name –locality voucher (Herbarium); haplotype of plastid *trnL-F*; allele of nuclear *AK1*.

**Deparia × nanakuraensis K.Hori**– JAPAN. Akita pref., Noshiro city, Futatsui town, Nanakura-shrine, 23m alt., 40°12'9.48"N, 140°15'29.82"E, 7 Jul 2020, *K. Hori 3391* (MAK, MBK); MT898446 (*trnL-F*); MT887301, MT887307 (*AK1*). ibid., *K. Hori 3392* (MAK, MBK); MT898447 (*trnL-F*); MT887302, MT887308 (*AK1*). ibid., *K. Hori 3393* (MAK, MBK); MT898448 (*trnL-F*); MT887303, MT887309 (*AK1*).

***D. pterorachis* (Christ) M.Kato**– JAPAN. Hokkaido Pref., Sapporo city, Minamiku, Jouzannkei, 530m alt., 42°55'36.8"N, 141°10'6.1"E, July 30 2018, *K. Hori 3053* (MBK); MT898441 (*trnL-F*); MT887299 (*AK1*). ibid., Ebetsu city, Nopporo nature park, July 30 2018, *K. Hori 3054* (MBK); MT898442 (*trnL-F*); MT887300 (*AK1*). ibid., *K. Hori 3055* (MBK); MT898443 (*trnL-F*); LC421964 (*AK1*, Hori 2018).

***D. viridifrons* (Makino) M.Kato**– JAPAN. Kochi pref., Takaoka county, Ochi town, Mt. Yokogura, May 30 2018, *K. Hori 2971* (MBK); LC421960 (*trnL-F*, Hori and Murakami 2019); LC468191 (*AK1*, Hori 2018). ibid., Oct 17 2018, *K. Hori 3060* (MAK); MT898444 (*trnL-F*); MT887305 (*AK1*). ibid., Oct 17 2018, *K. Hori 3061* (MAK); MT898445 (*trnL-F*); MT887306 (*AK1*).

***D. coreana* (Christ) M.Kato**– JAPAN. Aomori Pref., Kamikita county, Shichinohe town, Jul 26 2018, *Hori 3047* (MBK); MW051518 (*trnL-F*); MW051522, MW051523 (*AK1*).

***D. henryi* (Baker) M.Kato**– JAPAN. Kyoto Pref., Kyoto City, Jul 14 2018, *Hori 3028* (MBK); MW051514 (*trnL-F*); MW051527, MW0515278, MW051529 (*AK1*).

***D. okuboana* (Makino) M.Kato**– JAPAN. Kyoto pref., Kyoto city, Jul 14 2018, *Hori 3033* (MBK); MW051515 (*trnL-F*); MW051530, MW051531 (*AK1*).

**D. pycnosora (Christ) M.Kato var. albosquamata M.Kato**– JAPAN. Nagano Pref., Nagano city, Togakushi shrine, Okusha, Jul 9 2020, *K. Hori 3382* (MAK); MW051519 (*trnL-F*); MW051520, MW051521 (*AK1*).

**D. pycnosora (Christ) M. Kato var. mucilagina M.Kato**– JAPAN. Nagano Pref., Nagano city, Togakushi shrine, Okusha, Jul 9 2020, *K. Hori 3380* (MAK); MW051516 (*trnL-F*); MW051526 (*AK1*).

**D. pycnosora (Christ) M. Kato var. pycnosora M.Kato**– JAPAN. Aomori Pref., Kamikita county, Touhoku town, Otsutomo, Jul 26 2018, *K. Hori 3052* (MAK); MW051517 (*trnL-F*); MW051524, MW051525 (*AK1*).

***D. unifurcata* (Baker) M.Kato**– JAPAN. Kyoto Pref., Kyoto city, Jul 14 2018, *K. Hori 3029* (MBK); LC468192 (*trnL-F*, Hori and Murakami 2019); LC421961, LC421962 (*AK1*, Hori 2018).
